# Cellular Immunity Confers Transient Protection in Experimental Buruli Ulcer following BCG or Mycolactone-Negative *Mycobacterium ulcerans* Vaccination

**DOI:** 10.1371/journal.pone.0033406

**Published:** 2012-03-08

**Authors:** Alexandra G. Fraga, Teresa G. Martins, Egídio Torrado, Kris Huygen, Françoise Portaels, Manuel T. Silva, António G. Castro, Jorge Pedrosa

**Affiliations:** 1 Life and Health Sciences Research Institute (ICVS), School of Health Sciences, University of Minho, Braga, Portugal; 2 ICVS/3B's - PT Government Associate Laboratory, Braga/Guimarães, Portugal; 3 Scientific Service Immunology, Scientific Institute of Public Health WIV-ISP (Site Ukkel), Brussels, Belgium; 4 Mycobacteriology Unit, Department of Microbiology, Institute of Tropical Medicine, Antwerp, Belgium; 5 Institute for Molecular and Cell Biology, Porto, Portugal; University of Padova, Medical School, Italy

## Abstract

**Background:**

Buruli ulcer (BU) is an emerging infectious disease caused by *Mycobacterium ulcerans* that can result in extensive necrotizing cutaneous lesions due to the cytotoxic exotoxin mycolactone. There is no specific vaccine against BU but reports show some degree of cross-reactive protection conferred by *M. bovis* BCG immunization. Alternatively, an *M. ulcerans*-specific immunization could be a better preventive strategy.

**Methodology/Principal Findings:**

In this study, we used the mouse model to characterize the histological and cytokine profiles triggered by vaccination with either BCG or mycolactone-negative *M. ulcerans*, followed by footpad infection with virulent *M. ulcerans*. We observed that BCG vaccination significantly delayed the onset of *M. ulcerans* growth and footpad swelling through the induction of an earlier and sustained IFN-γ T cell response in the draining lymph node (DLN). BCG vaccination also resulted in cell-mediated immunity (CMI) in *M. ulcerans*-infected footpads, given the predominance of a chronic mononuclear infiltrate positive for iNOS, as well as increased and sustained levels of IFN-γ and TNF. No significant IL-4, IL-17 or IL-10 responses were detected in the footpad or the DLN, in either infected or vaccinated mice. Despite this protective Th1 response, BCG vaccination did not avoid the later progression of *M. ulcerans* infection, regardless of challenge dose. Immunization with mycolactone-deficient *M. ulcerans* also significantly delayed the progression of footpad infection, swelling and ulceration, but ultimately *M. ulcerans* pathogenic mechanisms prevailed.

**Conclusions/Significance:**

The delay in the emergence of pathology observed in vaccinated mice emphasizes the relevance of protective Th1 recall responses against *M. ulcerans*. In future studies it will be important to determine how the transient CMI induced by vaccination is compromised.

## Introduction

Buruli ulcer (BU), a neglected tropical disease caused by infection with *Mycobacterium ulcerans*, initially starts off as a nonulcerative cutaneous lesion that can eventually progress into an ulcer if left untreated. BU lesions are mainly due to the cytotoxic properties of the toxin secreted by *M. ulcerans*, known as mycolactone [1].

BU has become an emerging health problem in many endemic countries and the escalating morbidity rate has an overwhelming socioeconomic burden on the affected populations [2]. BU mainly affects rural populations with limited geographic and financial access to health services. As a result, medical-care seeking is delayed until advanced clinical forms of the disease are presented, including extensive skin destruction, multifocal lesions, and bone involvement [3], [4], [5], [6].

Currently, there is no vaccine against *M. ulcerans* infection and the classical approach for BU treatment has been surgical, involving wide excision of necrotic areas and surrounding healthy tissues [7], [8], [9]. Recently, the World Health Organization has recommended the use of rifampicin and streptomycin during an 8-week period to minimize the extent of surgery and decrease the recurrence rate [10]. Despite the progress made in early detection and treatment of BU, an efficient vaccine would be the best approach for the control of this devastating infectious disease.

As previously discussed, despite the production of the mycolactone exotoxin, *M. ulcerans* behaves like other pathogenic mycobacteria and has been shown to induce cell-mediated immunity (CMI) [11], [12], [13]. Moreover, efficient differentiation/proliferation of mycobacteria-specific T cells occurs in the draining lymph node (DLN) of *M. ulcerans*-infected mice and these effector T cells were found to be recruited to the infectious focus [13], where they can mediate bacterial control by enhancing macrophage antimicrobial mechanisms through the secretion of IFN-γ [14].

However, despite the early development of T cell immunity in mouse experimental infections, the host response is not sufficient to inhibit the proliferation of highly virulent *M. ulcerans* strains [13]. The associated increase in bacterial loads and consequent mycolactone build-up results in the destruction of cells recruited to the infection focus [13], [15], inhibition of the early IFN-γ-dependent activation of macrophages [14], and tissue necrosis as well as in destruction of the DLN itself [13], impairing therefore a sustained protective immune response.

The above mentioned findings suggest that vaccination protocols triggering a heightened specific CMI response could play a role in BU prevention. In line with this rationale, both experimental studies and clinical trials are consistent with BCG vaccination conferring a significant, although transient, protection against BU lesions [16], [17], [18], [19], [20], [21], [22], [23]. It has also been suggested that homologous vaccination inducing *M. ulcerans* specific immune responses, could elicit a more appropriate/prolonged protective immunity against *M. ulcerans* infection [21], [23].

Therefore, unraveling protective immunological mechanisms against *M. ulcerans* induced by immunization with mycobacterial antigens is necessary for the development of improved vaccination strategies. In the present study we characterized the dynamics of the local and DLN immune responses of mice previously immunized with BCG or with a mycolactone-negative strain of *M. ulcerans* and later infected with a highly virulent strain of *M. ulcerans*.

## Methods

### Bacteria

*M. bovis* BCG Pasteur was obtained from the Trudeau Institute Mycobacterium collection and grown in Proskauer Beck medium containing 0.05% Tween 80 to mid-log phase and frozen in aliquots at −80°C. *M. ulcerans* strains were selected from the Institute of Tropical Medicine (ITM) collection in Antwerp, Belgium. *M. ulcerans* 5114 is a mycolactone-negative strain due to repeated sub-culturing, leading to the spontaneous loss of MUP038 encoding the type II thioesterase, the acyltransferase domain of module 5 in *mlsA1* and the acyltransferase domain of modules 1 and 2 in *mlsB*, genes that are involved in the synthesis of mycolactone [24], [25]. *M. ulcerans* strain 98–912 is described to be highly virulent for mice [15] and produces mycolactone type D [26]. The isolates were grown on Middlebrook 7H9 medium (Becton, Dickinson and Company) with 1.5% of agar at 32°C for approximately 6–8 weeks. For the preparation of the inoculum, *M. ulcerans* was recovered, diluted in phosphate-buffered saline (PBS) to a final concentration of 1 mg/ml and vortexed using glass beads.

### Immunization and infection protocol

Eight-week-old female BALB/c mice were obtained from Charles River (Barcelona, Spain) and were housed in specific pathogen free conditions with food and water *ad libitum*. Mice were either not vaccinated or vaccinated with a subcutaneous injection of 5–6 log_10_ CFU of *M. bovis* BCG or *M. ulcerans* 5114, two months before *M. ulcerans* 98–912 infection. Infection was carried out in the left hind footpad with 0.03 ml of *M. ulcerans* suspension containing either 4 log_10_ or 2 log_10_ CFU, as indicated. The right hind footpad was used as a control. Footpad swelling was monitored throughout the experimental infection by using a caliper to measure the diameter of the frontal area of the footpad. The time to develop relevant footpad swelling, which was considered to be at 2.60 mm, was shown by Kaplan-Meier analysis. After emergence of swelling, mice from all groups progressed to ulceration of the footpad. For ethical reasons, mice were sacrificed upon onset of footpad ulceration.

### Flow Cytometry

For intracellular staining, single cell suspensions of the popliteal lymph nodes were stimulated with 50 ng/ml of Phorbol Myristate Acetate (PMA) and 500 ng/ml of ionomycin in the presence of 10 ug/ml of Brefeldin A for 4 h at 37°C with 5% CO_2_. After this incubation period, cells were fixed and permeabilized. The presence of intracellular IFN-γ was determined by using anti-IFN-γ (clone XMG 1.2, BD Pharmingen). Cells were further labeled with a combinations of fluorochrome-labeled monoclonal antibodies specific for CD3 (clone 145-2C11), CD19 (clone 1D3), CD4 (clone RM4-5) or CD8 (clone 53–6.7). Cells were analysed using CELLQuest software on a Becton Dickinson FACSCalibur flow cytometer.

### Extraction of cells from infected footpads

Infected footpads were collected and incubated in 125 U/ml collagenase XI (Sigma-Aldrich) diluted in DMEM (Gibco), for 2 h at 37°C. Each footpad was filtered through a 40 µm nylon cell strainer and washed with DMEM supplemented with 10% heat-inactivated fetal bovine serum (Sigma-Aldrich).

### Bacterial load determination

Infected footpads were excised, individually homogenized and diluted in 2 ml of PBS. The supernatant was recovered and decontaminated with 3 ml of sterile HCl 1M supplemented with 0,001% phenol red. After an incubation period of 15 min at room temperature the suspension was neutralized with sterile NaOH 1M and centrifuged for 20 min. The supernatant was discarded and the pellet resuspended in PBS. Serial dilutions of the suspension were plated on nutrient 7H9 agar. Bacterial colony formation was counted after 6–8 weeks of incubation at 32°C.

### Real Time PCR

Cell suspensions from the infected footpad were frozen in TRIzol reagent (Invitrogen), and total RNA was extracted according to the manufacturer's protocol. Reverse transcription was done with whole RNA using SuperScript II (Invitrogen) and Oligo(dT) (Invitrogen) according to the manufacturer's instructions. cDNA was amplified and the Biorad C1000 real-time PCR was used for quantification of Ubiquitin (UBQ), IFN-γ, IL-4, IL-10, IL-17, TNF, and MIP2 mRNA with Sybr Green (Qiagen). The primer sequences were designed and synthesized by TIB MolBiol and were as follows: UBQ forward, TGG CTA TTA ATT ATT CGG TCT GCA T; UBQ reverse, GCA AGT GGC TAG AGT GCA GAG TAA; IFN-γ forward TGG CAA AAG GAT GGT GAC ATG; IFN-γ reverse GAC TCC TTT TCC GCT TCC TGA; IL-4 forward, CTC ATG GAG CTG CAG AGA CTC TT; IL-4 reverse, CAT TCA TGG TGC AGC TTA TCG A; IL-10 forward, TTT GAA TTC CCT GGG TGA GAA; IL-10 reverse, GCT CCA CTG CCT TGC TCT TAT T; IL-17 forward, CTC AGA CTA CCT CAA CCG TTC CA; IL-17 reverse, TTC CCT CCG CAT TGA CAC A; TNF forward, GCC ACC ACG CTC TTC TGT CT; TNF reverse, TGA GGG TCT GGG CCA TAG AAC; MIP2 forward, CTC AGT GCT GCA CTG GT; and MIP2 reverse, AGA GTG GCT ATG ACT TCT GTC T.

### Histological studies

Mouse popliteal lymph nodes and footpads were harvested, fixed in buffered formalin and embedded in paraffin. Light-microscopy studies were performed on tissue sections stained with hematoxylin and eosin (HE) or Ziehl-Neelsen (ZN). For immunofluorescence staining, tissue sections were deparaffinised and hydrated. Antigen retrieval was performed with Citrate Buffer (Lab Vision Corporation) at 96°C for 20 min. Unspecific binding was prevented by incubating with 5% FBS in PBS with 0,1% Tween 20 and Triton X 100 (Sigma-Aldrich) for 1 h at room temperature, after which purified rabbit polyclonal anti-NOS2 (N-20) (Santa Cruz Biotechnology) was added to the section at a concentration of 1∶100 followed by overnight incubation at 4°C. Goat anti-rabbit AlexaFluor 568 secondary antibody (Molecular Probes) was added at a concentration of 1∶500 in blocking solution for 1 hour at room temperature. DAPI (4¢,6-diamido-2-phenylindole hydrochloride, Molecular Probes) was used to detect nuclei. Images were obtained with an Olympus BX61 microscope.

### Statistical analysis

Differences between the means of experimental groups were analyzed with the two-tailed Student's t-test. Differences with a *P* value≤0.05 were considered significant.

### Ethics Statement

This study was approved by the Portuguese national authority for animal experimentation Direcção Geral de Veterinária (ID: DGV 594 from 1^st^ June 2010). Animals were kept and handled in accordance with the guidelines for the care and handling of laboratory animals in the Directive 2010/63/EU of the European Parliament and of the Council.

## Results

### Immunization with BCG induces an earlier and stronger Th1 response in the footpad and the DLN, delaying the onset of pathology caused by highly virulent *M. ulcerans*

**Figure 1:**
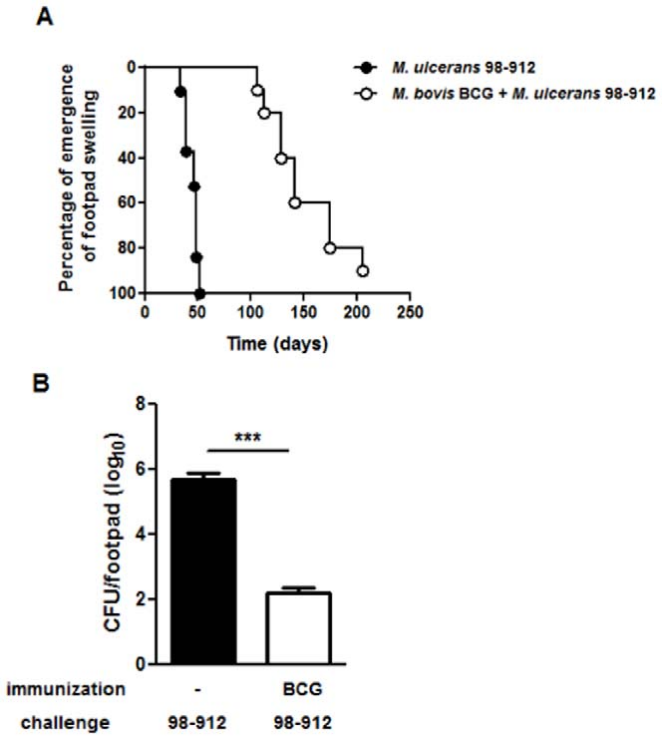
Immunization with BCG delays the onset of footpad swelling and reduces bacillary growth in *M. ulcerans* infected mice. Mice were either non-immunized (•) or immunized with BCG (○) two months before challenge. All mice were infected in the footpad with 4 log_10_ CFU of *M. ulcerans* 98–912. (A) Footpad swelling was monitored throughout the experimental infection (*n* = 10). The onset of footpad swelling was considered at 2.60 mm, after which mice progressed to ulceration of the footpad. For ethical reasons, mice were sacrificed upon onset of footpad ulceration. (B) Infected footpads of BCG immunized mice (white bar) or non-immunized mice (black bar) were harvested at day 52 post-infection and processed for CFU count. Bars represent the mean ± SEM (*n* = 5) log_10_ CFU/footpad. Statistical significance was calculated with Student's *t* test (***p<0.001).

Understanding the mechanisms underlying the partial protection conferred by BCG vaccination may provide clues on how to design new interventions to improve host defensive immunity. Therefore, mice were immunized or not with BCG and infected two months later in the footpad with 4 log_10_ CFU of a highly virulent strain of *M. ulcerans*. In the non-vaccinated mouse group, the median time to develop swelling was 47 days, while in BCG vaccinated mice it was 142 days (Figure 1A). However, despite vaccination, footpads of mice of all experimental groups progressed to ulceration (data not shown). Confirming previous findings by Tanghe *et al* [27], prior BCG immunization was responsible for a reduction in the number of viable bacilli in the infected tissue, during which macroscopic lesions were absent in the footpads of BCG-vaccinated mice [18], [21], [22], with a 4 log_10_ reduction observed at day 52 post-infection (Figure 1B).

**Figure 2:**
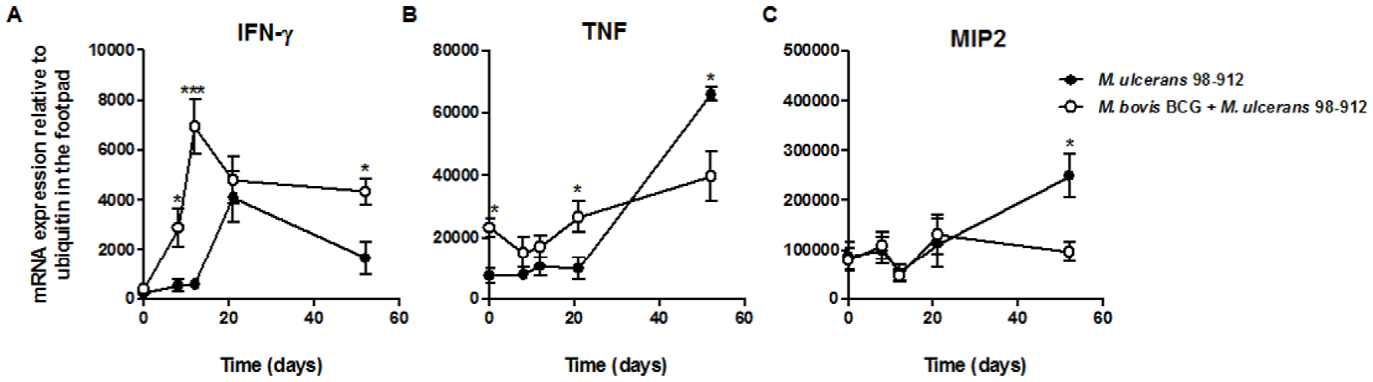
BCG vaccination induces an early Th1 cytokine profile in *M. ulcerans*-infected footpads. Mice were either non-immunized (•) or immunized with BCG (○) two months before challenge. All mice were infected in the footpad with 4 log_10_ CFU of *M. ulcerans* 98–912. At different times post-infection, total RNA from the footpad was extracted and the presence of mRNA for IFN-ã (A), TNF (B), MIP2 (C) was assessed by real-time PCR. Data points represent the mean ± SEM (*n* = 5–8) for each time point. Statistical significance was calculated with Student's *t* test (*p<0.05; **p<0.01; ***p<0.001).

To characterize the type of immune response associated with the BCG-induced delay of *M. ulcerans* disease, we carried out a comparative analysis of cytokine kinetics in footpads. As previously described, in the footpads of non-immunized *M. ulcerans*-infected mice [14], we found an increase in the mRNA levels of the Th1 cytokine IFN-γ during the first weeks of infection, which waned towards the end of the experiment when the infectious process was advanced (Figure 2A). Immunization with BCG resulted in an earlier, more intense and sustained expression of IFN-γ (Figure 2A). Regarding the expression of the pro-inflammatory cytokine TNF, non-immunized mice only revealed a significant increase by day 52 post-infection (Figure 2B), while BCG-immunized mice showed high mRNA levels of TNF at earlier time points that were maintained throughout the experimental period (Figure 2B). Analysis of MIP2 expression revealed a peak in the mRNA level in the footpads of non-immunized mice at day 52 post-infection (Figure 2C). In comparison, no significant differences were observed in BCG-immunized mice over the course of infection (Figure 2C). The expression of the Th2 cytokine IL-4, the Th17 cytokine IL-17, as well as of IL-10, was low and did not vary during infection regardless immunization (Figure S1).

**Figure 3:**
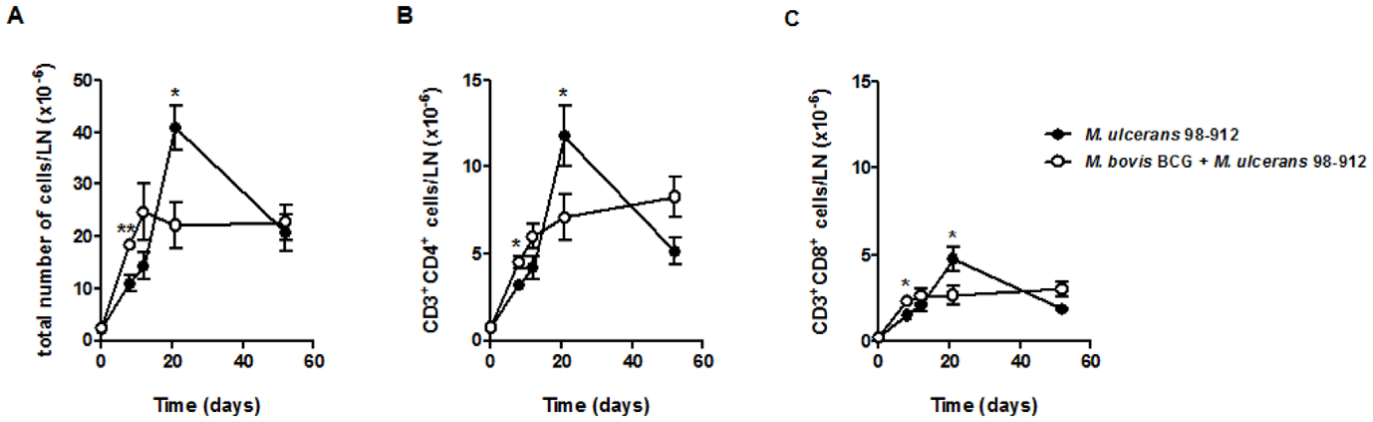
Vaccination with BCG induces an early CD4^+^ T cell response in the DLN of *M. ulcerans*-infected mice. Mice were either non-immunized (•) or immunized with BCG (○) two months before challenge. All mice were infected in the footpad with 4 log_10_ CFU of *M. ulcerans* 98–912. At different time points post-infection, the total number of leukocytes in the popliteal lymph node of the infected footpad was counted (A) and the number of CD4^+^ (B), and CD8^+^ (C) cells was determined by flow cytometry. The data points represent the mean ± SEM (*n* = 5–8). Statistical significance was calculated with Student's *t* test (*p<0.05; **p<0.01).

Having observed that the delay in both swelling and bacterial proliferation in the footpads of BCG-vaccinated mice (Figure 1) correlated with a Th1 cytokine profile (Figure 2), we next studied the immunological events taking place in the DLN, where the initiation of the adaptive immune response against *M. ulcerans* occurs [13]. In the DLN of non-immunized mice, infection with *M. ulcerans* induced a significant peak in the total number of cells at day 21 post-infection (Figure 3A), including CD4^+^ and CD8^+^ cells (Figure 3B and C), which later declined with the progression of the infectious process, as previously described [13]. BCG vaccination induced a distinct pattern in the DLN cellular dynamics, with an earlier increase in the numbers of total, CD4^+^ and CD8^+^ cells, levels that were maintained during the experimental infection.

**Figure 4:**
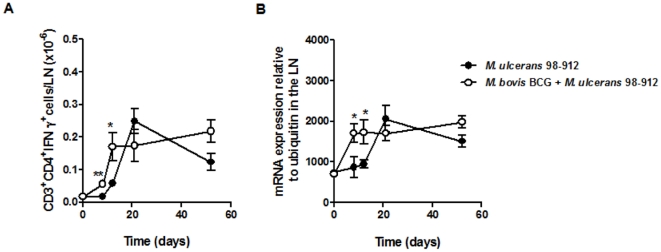
Vaccination with BCG induces an early IFN-γ response in the DLN of *M. ulcerans*-infected mice. Mice were either non-immunized (•) or immunized with BCG (○) two months before challenge. All mice were infected in the footpad with 4 log_10_ CFU of *M. ulcerans* 98–912. (A) At different time points post-infection, the total number of CD3^+^ CD4^+^ IFN-γ^+^ in the DLN of the infected footpad was determined by flow cytometry. The data points represent the mean ± SEM (*n* = 5–8). (B) At different times post-infection, total RNA from the popliteal lymph node was extracted and the presence of mRNA for IFN-γ was assessed by real-time PCR. Data points represent the mean ± SEM (*n* = 5–8) for each time point. Statistical significance was calculated with Student's *t* test (*p<0.05). Statistical significance was calculated with Student's *t* test (*p<0.05; **p<0.01).

The early appearance of CD4^+^ T cells in the DLN of BCG-vaccinated mice prompted us to investigate whether these lymphocytes were able to mount an adaptive effector response. For this, we followed the kinetics of IFN-γ-producing CD4^+^ cells in the DLN by intracellular cytokine flow cytometry (Figure 4A), as well as the mRNA expression of IFN-γ (Figure 4B), IL-4, and IL-10 (Figure S2) by real-time PCR. In non-immunized mice, we observed a significant increase in the IFN-γ response in the DLN at day 21 post-infection, which gradually declined (Figure 4A and B). In comparison, BCG-vaccinated mice showed an increased IFN-γ response in the DLN as early as 8 days post-infection, a level which was maintained throughout the experimental infection (Figure 4A and B). No relevant IL-4 or IL-10 responses were found in the DLN, as the levels of these cytokines mRNA, in both non-immunized and BCG-immunized mice, were low and did not vary throughout the infection period (Figure S2).

### Regardless of *M. ulcerans* challenge dose, immunization with BCG only confers partial protection, associated with a transient mononuclear infiltrate with activated macrophages

**Figure 5:**
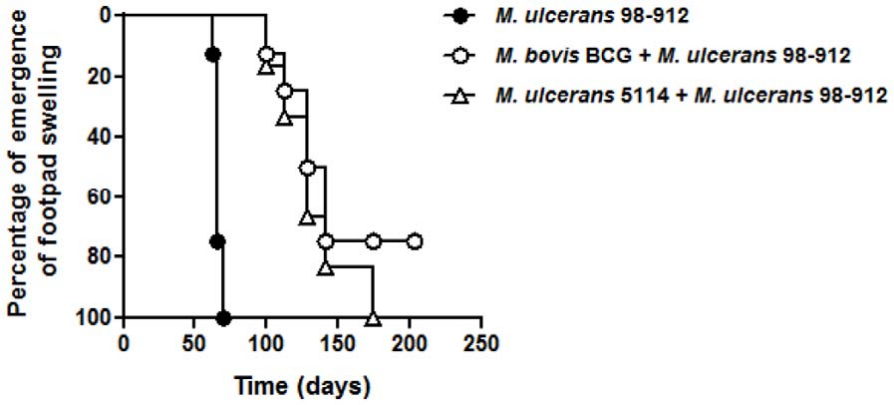
Immunization with BCG or mycolactone-negative *M. ulcerans* delays the onset of footpad swelling after challenge with a low dose of virulent *M. ulcerans*. Mice were either non-immunized (•) or immunized with BCG (○) or with *M. ulcerans* 5114 (▵) two months before challenge. All mice were infected in the footpad with 2 log_10_ CFU of *M. ulcerans* 98–912. Footpad swelling was monitored throughout the experimental infection (*n* = 10). Initial footpad swelling was considered at 2.60 mm, after which mice progressed to ulceration of the footpad. For ethical reasons, mice were sacrificed upon onset of footpad ulceration.

Despite the development of an earlier and stronger Th1 response associated with the initial protection conferred by vaccination, mice eventually developed footpad swelling (Figure 1), followed by ulceration (data not shown), indicating that progressive disease was significantly delayed but not prevented. It has been previously suggested that high doses of *M. ulcerans* inocula could compromise the BCG-induced protection [23]. However, even when a lower challenge dose of *M. ulcerans* (2 log_10_ CFU) was tested, we still observed that vaccination only conferred partial protection, as assessed by the emergence of footpad swelling (Figure 5).

**Figure 6:**
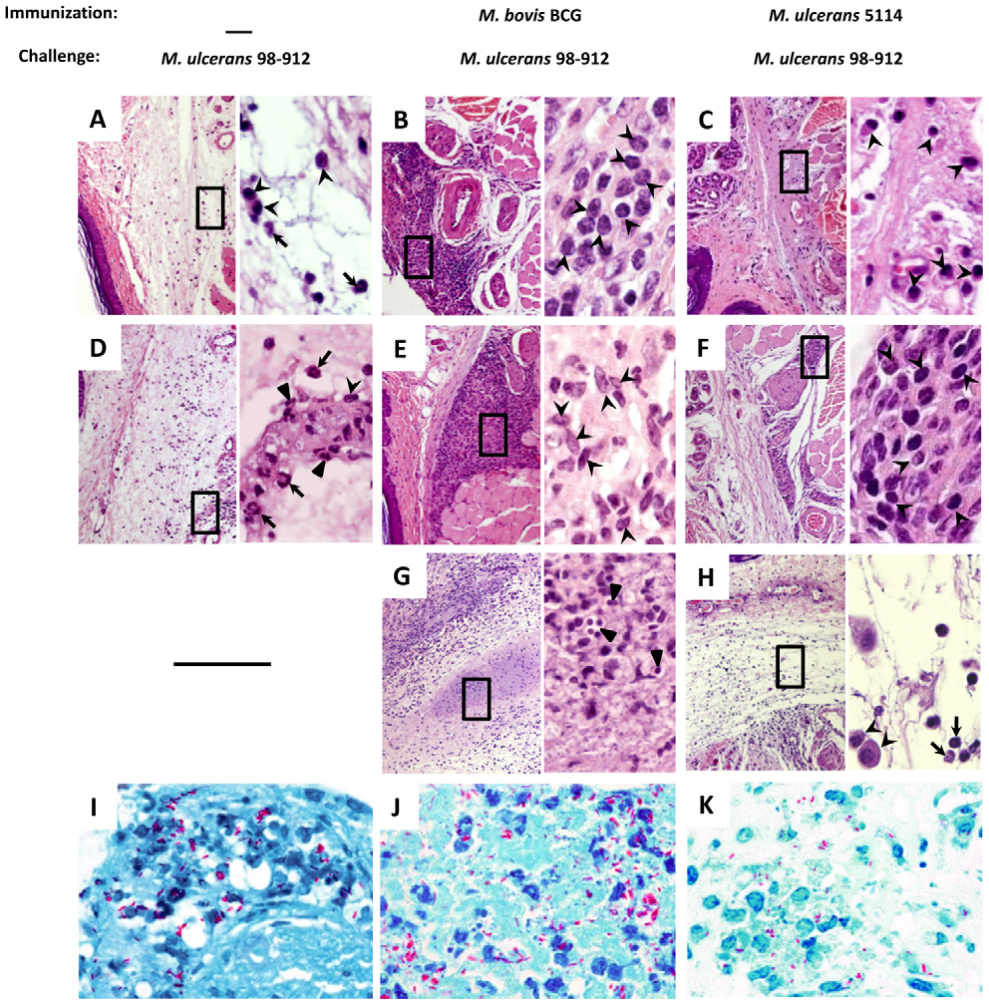
Immunization induces a transient mononuclear inflammatory response in the footpad of *M. ulcerans* challenged mice. Mice were either non-immunized (A, D, and I) or immunized with BCG (B, E, G, and J) or with mycolactone-negative *M. ulcerans* 5114 (C, F, H, K) two months before challenge. All mice were infected in the footpad with 2 log_10_ CFU of *M. ulcerans* 98–912. The footpads were harvested at on day 60 (A–C), day 70 (D–F and I), or day 150–200 (G–H and J–K) and processed for histology. Tissue sections were stained with HE (A–H) or ZN (I–K). (A, D, and I) Non-immunized mice showed a persistent mixed inflammatory infiltrate composed of mononuclear cells (arrowheads) and neutrophils (arrows). Necrotic areas harbored abundant extracellular bacilli (I) and cells with apoptotic morphology (triangles). (B, C, E, F, G, H, J, and K) The footpads of vaccinated mice presented a mononuclear inflammatory infiltrate (arrowheads) throughout the initial weeks of experimental infection, which was later replaced by a predominantly neutrophilic infiltrate (arrows) and/or apoptosis (triangles) and extracellular bacilli (J, K). Images are representative of 4 footpads per group. Original magnification: 10×. Original magnification of HE inserts: 100×. Original magnification of ZN: 100×.

**Figure 7:**
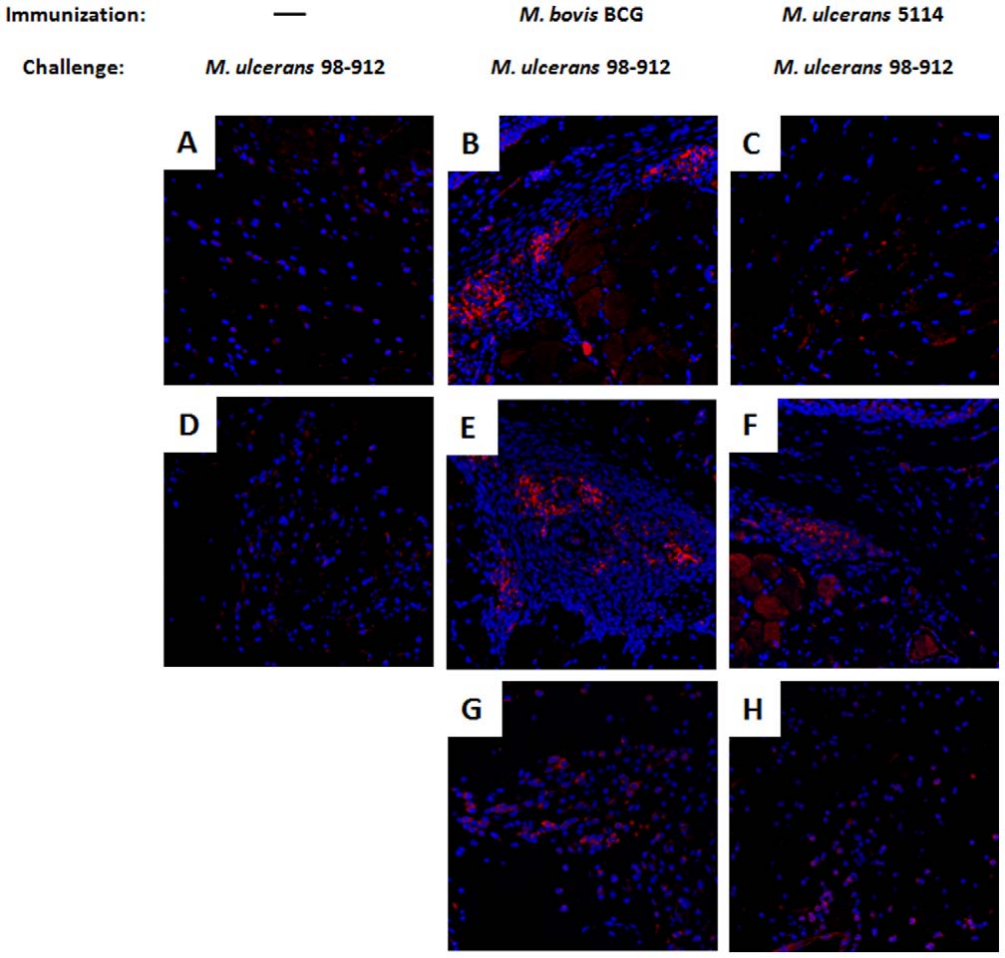
Immunization increases iNOS positivity in the footpad of *M. ulcerans* challenged mice. Mice were either non-immunized (A and D) or immunized with BCG (B, E, and G) or with mycolactone-negative *M. ulcerans* 5114 (C, F, and H) two months before challenge. All mice were infected in the footpad with 2 log_10_ CFU of *M. ulcerans* 98–912. The footpads were harvested at on day 60 (A–C), day 70 (D–F), or day 100–200 (G–H and J–K) and processed for histology. Cells were stained for the presence of iNOS (red) and nuclei were stained with DAPI (blue). In footpads of non-immunized mice, scarce or no iNOS positive cells were found in the tissue (A, D). The footpads of vaccinated mice presented foci of iNOS positive cells during the initial weeks of infection (B–C and E–F). With progression of infection, iNOS positivity became sparse. Images are representative of 4 footpads per group. Original magnification: 20×.

This transitory period of protective immunity correlated with a differential histological pattern in the infected footpads (Figure 6). We found a strong cellular response in BCG-vaccinated mice during the first 70 days of *M. ulcerans* infection (Figure 6B and E), predominantly consisting of mononuclear cells and granuloma-like organizations (Figure 6B and E inserts). These areas of inflammatory infiltrate showed numerous and large foci, positive for inducible nitric oxide synthase (iNOS) (Figure 7B and E), an enzyme involved in the production of nitric oxide, which is necessary for mycobacterial killing [28], [29], [30]. In accordance, bacilli were rarely found in the subcutaneous tissue during this initial period of immune-mediated protection (data not shown). However, with the progression of the infectious process, the mononuclear inflammatory infiltrate was later replaced by areas of subcutaneous tissue damage, extensive necrosis (Figure 6G), and numerous extracellular bacilli (Figure 6J). Additionally, immunofluorescent staining for iNOS showed sparse positive cells throughout the tissue (Figure 7G).

In non-immunized infected mice, as previously described [15], this type of necrotic areas were seen much earlier, as shown at 60 days post-infection (Figure 6A), and expanded progressively into healthy tissues until day 70 (Figure 6D), when footpad ulceration emerged (data not shown). A persistent mixed inflammatory infiltrate was found at the periphery of the necrotic areas, composed by mononuclear cells and neutrophils (Figure 6A and D inserts), which were negative for iNOS staining (Figure 7A and D). These necrotic areas harbored abundant extracellular bacilli (Figure 6I) and cells with apoptotic morphology (Figure 6D insert).

**Figure 8:**
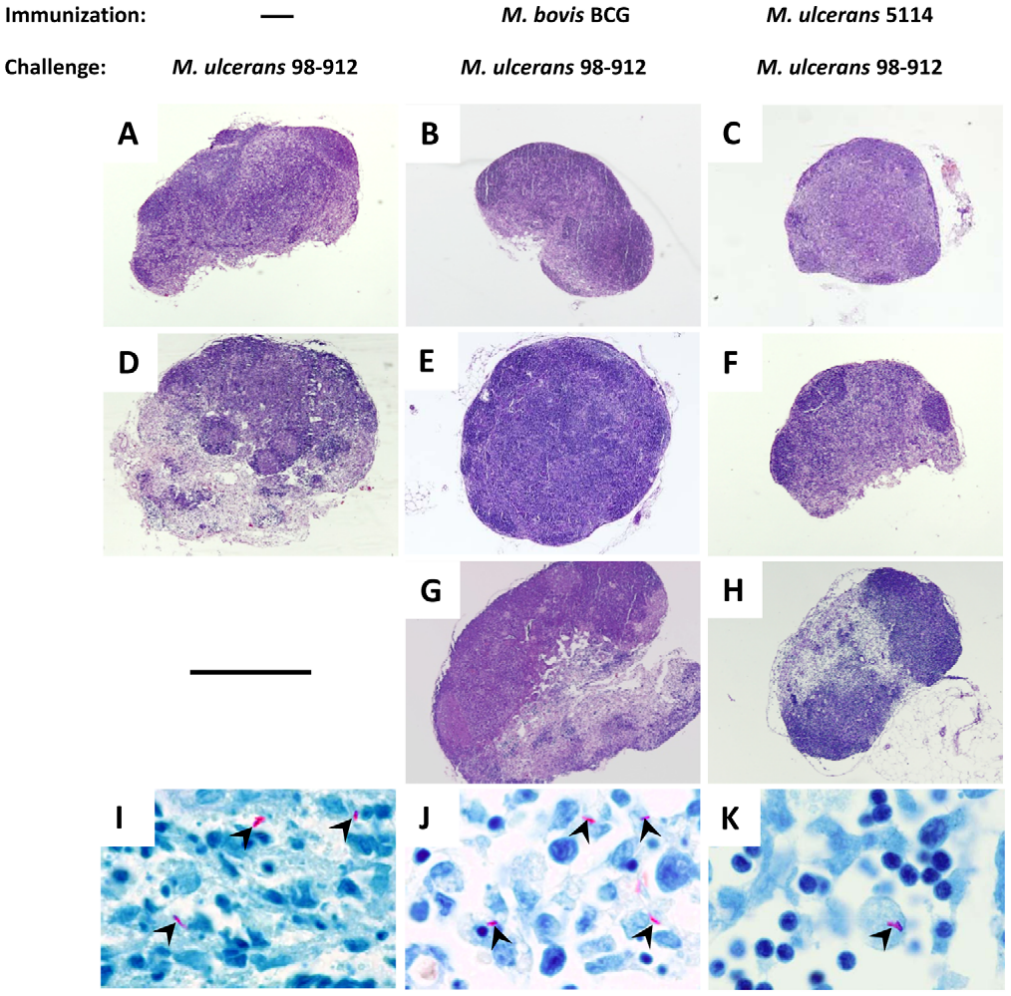
Immunization with BCG or mycolactone-negative *M. ulcerans* 5114 delays the destruction of the DLN during infection with virulent *M. ulcerans*. Mice were either non-immunized (A, D, and I) or immunized with BCG (B, E, G, and J) or with mycolactone-negative *M. ulcerans* 5114 (C, F, H, K) two months before challenge. All mice were infected in the footpad with 2 log_10_ CFU of *M. ulcerans* 98–912. The DLN were recovered at day 60 (A–C), day 70 (D–F and I), and days 100–200 (G–H and J–K) and processed for histology. Tissue sections were stained with HE (A–H) or ZN (I–K). (A, D, and I) In non-immunized mice infection resulted in extensive areas of necrosis and bacterial colonization by day 70 post-infection. (B, C, E, F, G, H, J, and K) Immunized mice showed no alterations in the structure of the DLN during these first 70 days. As the infectious process progressed in immunized mice, bacilli could be found throughout the tissue (arrowheads) along with signs of tissue destruction. Images are representative of 4 lymph nodes per group analyzed. Original magnification (A–H): 4×. Original magnification (I–K): 100×.

In addition, histological analysis of DLN from non-immunized mice revealed that *M. ulcerans* infection lead to severe structural damage during the first 70 days of infection, ultimately resulting in extensive areas of cell death, necrotic alterations, and bacterial colonization (Figure 8D and I), as recently reported in detail [13]. Conversely, BCG-immunized mice showed no alterations in the structure of the DLN during these first 70 days (Figure 8B and E). However, as the infectious process progressed, numerous bacilli were found along with signs of tissue destruction at 100–125 days post-infection (Figure 8G and J).

### Immunization with mycolactone-negative *M. ulcerans* 5114 transiently delays the onset of pathology induced by the highly virulent *M. ulcerans* strain

It has been suggested that homologous *M. ulcerans* vaccination could elicit a more appropriate/prolonged protective immunity [21], [23] than the one induced by BCG vaccination. Therefore, we decided to test the level of protection conferred by immunization with a live-attenuated *M. ulcerans* strain. For this, we immunized mice with the mycolactone-negative strain *M. ulcerans* 5114, generated by spontaneous mutation after successive *in vitro* passages [25], before challenging mice with 2 log_10_ of the highly virulent mycolactone-positive strain. Similar to the findings in BCG-vaccinated mice, immunization with mycolactone-negative *M. ulcerans* induced significant protection against the highly virulent strain, but failed to permanently prevent disease, with a median time to develop swelling of 129 days (Figure 5).

The histological analysis of infected footpads of mice vaccinated with *M. ulcerans* 5114 revealed scarce infiltrates at day 60 post-infection (Figure 6C and insert). By day 70 post-infection, a moderate chronic inflammatory infiltrate was observed (Figure 6F and insert) along with small foci of iNOS positive cells (Figure 7F). This infiltrate was eventually replaced by a predominantly neutrophilic infiltrate, edema, cell death (Figure 6H and insert), numerous extracellular bacilli (Figure 6K), and characterized by a loss of iNOS positivity (Figure 7H). As observed in BCG-vaccinated mice, immunization with strain 5114 resulted in DLN alterations only after day 70 post-infection (Figure 8H and I), with bacilli scattered throughout the destroyed lymphoid tissue (Figure 8J and K).

## Discussion

Currently, there is no specific vaccine against *M. ulcerans*. However, there is evidence in the literature that suggests the development of cross-reactive immunity with BCG during *M. ulcerans* infections. BCG is a live-attenuated strain of *M. bovis* used for the prevention of tuberculosis [31] and leprosy [32]. It has also been reported that BCG confers some degree of protection against severe, disseminated manifestations of *M. ulcerans* infection, namely osteomyelitis [19], [20], but only induces a transient protective activity against the development of cutaneous lesions. Indeed, clinical trials confirmed some efficacy of BCG vaccination at preventing the development of new BU lesions; however, this protection waned from 63–72% to nearly zero during the first year of the study [16], [17]. Additionally, studies in the murine model showed that prior immunization with BCG, or plasmid DNA encoding Ag85A from BCG, conferred some degree of protection against experimental *M. ulcerans* infection, although not sustained [18], [21], [22], [23], [27].

For experimental tuberculosis, it is known that BCG vaccination induces the generation of protective cellular memory and, upon subsequent challenge, accelerates the expansion of antigen-specific T cells, resulting in a significant delay in the establishment of infection [33]. We have recently shown that CMI is triggered during *M. ulcerans* infection. Specifically, early during experimental murine infection with virulent *M. ulcerans*, antigen-specific IFN-γ-producing T cells develop in the DLN and differentiated CD4^+^ cells migrate to the site of infection [13]. However, the subsequent increase in bacterial burdens and likely mycolactone accumulation eventually leads to the depletion of the recruited cells and to extensive destruction of the DLN, abrogating the initially triggered CMI [13]. Herein, we show that in experimental BU, vaccination with BCG, although not preventing the eventual emergence of *M. ulcerans* disease, significantly delayed its onset through the induction of an earlier and sustained IFN-γ T cell response in the DLN. In addition, vaccination resulted in increased CMI in *M. ulcerans*-infected footpads, as measured by a predominance of a mononuclear infiltrate, with the activation of macrophages as shown by areas of iNOS positivity, as well as increased and sustained levels of IFN-γ and TNF, but not IL-4, IL-10 or IL-17. These responses translated into an improved, albeit transitory, containment of bacterial proliferation. In line with these findings, it has been shown that IFN-γ, TNF, and iNOS play an important role in protective immunity against *M. ulcerans* experimental infections, contributing to control of bacterial proliferation [14], [28], [34]. Additionally, the significant levels of IFN-γ observed in BCG-immunized mice throughout *M. ulcerans* infection were associated with a low expression of both IL-4 and IL-17. Accordingly, it has been reported that IFN-γ negatively regulates the induction of both Th2 [35] and Th17 [36] subsets.

Despite inducing an earlier protective Th1 immune response, BCG vaccination did not prevent the ultimate progression of infection with highly virulent *M. ulcerans*, regardless of challenge dose. As suggested for tuberculosis, the partial protection conferred by BCG can be attributed to the fact that the BCG-induced immune response is quantitatively too weak to elicit sustained protection against *M. ulcerans* or, alternatively, is not functionally appropriate to stimulate the type of immune mechanisms required for persistent protection [37]. In either case, there would be continuous, although delayed, bacterial proliferation and mycolactone build-up, leading to the consequent impairment of protective cellular and molecular mechanisms. In future studies, it will be important to study later time points of infection in BCG-vaccinated mice, in order to determine how the partial protective immunological mechanisms induced by vaccination are compromised.

Species-specific vaccination strategies could also contribute to a more effective prevention of BU. Given the deficient cross-reactive immune response elicited by BCG vaccination during *M. ulcerans* infection, it has been argued that an *M. ulcerans*-specific immunization would be more efficient at controlling infection. Indeed, there are differences in genetic and antigenic mycobacterial composition that can result in variations of immunodominant epitopes [38]. Therefore, we evaluated the efficacy of a vaccination protocol with a mycolactone-negative strain of *M. ulcerans*. The genetic basis for mycolactone production is related to a highly unstable giant plasmid required for toxin biosynthesis [39] and deletion of plasmid sequences leads to a corresponding loss of toxin production [25]. Mycolactone-negative strains have been previously generated by spontaneous mutation after repeated *in vitro* passages or transposon mutagenesis attenuation [25]. In this study, we evaluated the efficacy of immunization with a live-attenuated vaccine based on the 5114 strain of *M. ulcerans* that lost the ability to produce mycolactone due to repeated *in vitro* passages [24], [25]. As for BCG, vaccination with this mycolactone-negative *M. ulcerans* only delayed the onset of pathology. This incomplete protective response may be related to the loss of important immunogenic epitopes, necessary to stimulate an adequate protective immune response, due to repeated *in vitro* passages. Accordingly, it has been hypothesized that continuous *in vitro* passages of *M. bovis* BCG resulted in deletions of immunodominant genes and further weakened the vaccine [40]. It is also worth mentioning that vaccination with another mycolactone-deficient *M. ulcerans* strain, genetically inactivated in the polyketide synthase *mlsB* gene, can confer sustained protection against *M. ulcerans* in an experimental mouse model (T. Einarsdottir, manuscript in preparation).

Our data show that BCG induces an immune response capable of transiently containing *M. ulcerans* proliferation, but ultimately fails at preventing the development of disease. Alternative and improved vaccination strategies could contribute to a more specific and effective response [38], [41]. For instance, the immunogenicity of conventional BCG or mycolactone-negative *M. ulcerans* could be improved by manipulation of its potential to generate protective immunity by overexpressing immunodominant antigens in recombinant vaccines, as previously described [42]. An alternative strategy would be the development subunit vaccines, in which an immunodominant mycobacterial protein in combination with appropriate adjuvant or live recombinant viral vector could be enough to induce sufficient protection. Another approach would be an anti-toxin based vaccine, since mycolactone has been described as being largely responsible for the immunopathology of *M. ulcerans* disease [1]. Such a vaccine could target either the biological activity of mycolactone by chemical modification or the enzymes involved in its synthesis [38]. In addition, it cannot be excluded that antibody-mediated immunity could also provide protection against *M. ulcerans*, with obvious implications for vaccine development [11], [41]. Different studies have detected a positive specific antibody response against *M. ulcerans* antigens in both BU patients and unaffected household contacts, but not in controls from regions where the disease is not endemic [43], [44], [45], [46]. Finally, it has also been suggested that inhabitants of BU endemic areas could be naturally immunized if repeatedly bitten by uninfected *Naucoris cimicoides* [47], a protective mechanism suggested for leishmaniasis [48] and Lyme disease [49]. Experimental studies showed that the pre-exposure of mice to insect salivary antigens rendered mice more resistant to infection through bites of *M. ulcerans* harboring-insects. However, the overall relevance and contribution of biting by *M. ulcerans*-colonized insects to the transmission of BU is unknown [50].

Collectively, our results support the perspective that the development of improved and/or specific vaccine strategies leading to a stronger and earlier CMI recall immune response against *M. ulcerans*, resulting in macrophage activation, before the build-up of mycolactone compromises T cell persistence, should be further explored. Moreover, our findings emphasize the importance of understanding how protective cellular immunity is compromised by highly virulent *M. ulcerans* and how the pathogenic effect could be overcome in order to develop vaccination strategies for efficient prophylaxis of BU.

## Supporting Information

Figure S1 **BCG vaccination induces an early Th1 cytokine profile in** ***M. ulcerans*** **-infected footpads.** Mice were either non-immunized (•) or immunized with BCG (○) two months before challenge. All mice were infected in the footpad with 4 log_10_ CFU of *M. ulcerans* 98–912. At different times post-infection, total RNA from the footpad was extracted and the presence of mRNA for IL-4 (A), IL-10 (B), and IL-17 (C) was assessed by real-time PCR. Data points represent the mean ± SEM (*n* = 5–8) for each time point. Statistical significance was calculated with Student's *t* test (*p<0.05; **p<0.01; ***p<0.001).
(TIF)

Figure S2 **Vaccination with BCG induces an early Th1 cytokine profile in the DLN of** ***M. ulcerans*** **-infected mice.** Mice were either non-immunized (•) or immunized with BCG (○) two months before challenge. All mice were infected in the footpad with 4 log_10_ CFU of *M. ulcerans* 98–912. At different times post-infection, total RNA from the popliteal lymph node was extracted and the presence of mRNA for IL-4 (A) and IL-10 (B) was assessed by real-time PCR. Data points represent the mean ± SEM (*n* = 5–8) for each time point. Statistical significance was calculated with Student's *t* test (*p<0.05).
(TIF)
