# Supplementary figures and images for: Cellular Immunity Confers Transient Protection in Experimental Buruli Ulcer following BCG or Mycolactone-Negative Mycobacterium ulcerans Vaccination
Source: PLoS One. 2012 Mar 8;7(3):e33406. doi: 10.1371/journal.pone.0033406 (PMC3297633; doi:10.1371/journal.pone.0033406)

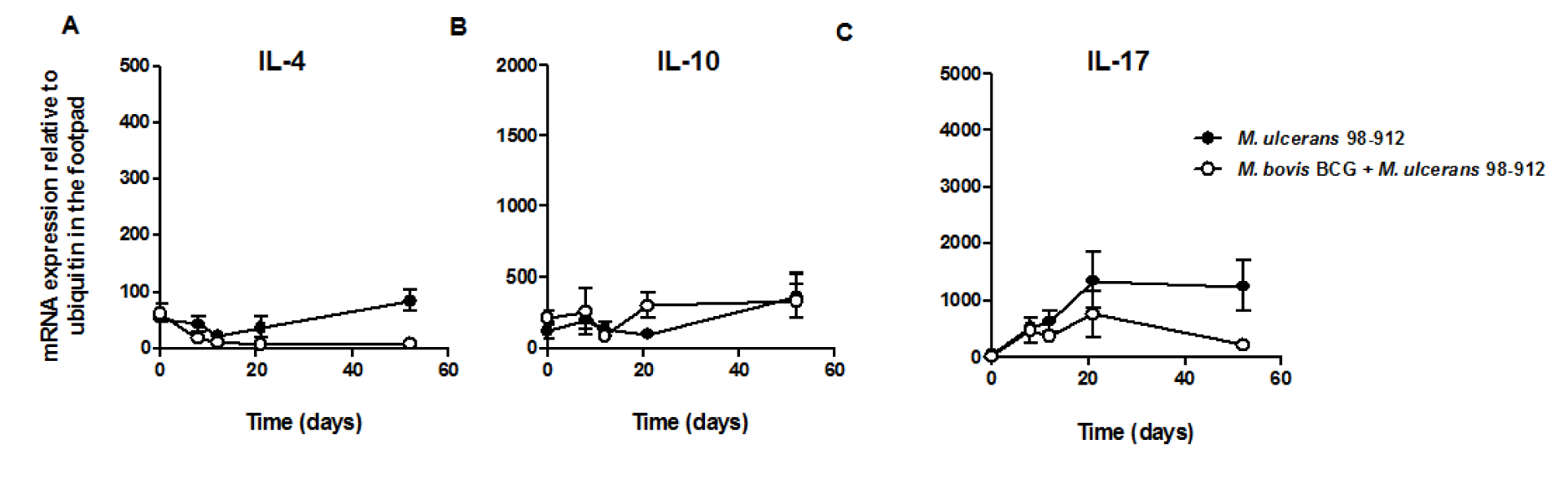

Supplement: Figure S1 — BCG vaccination induces an early Th1 cytokine profile in M. ulcerans -infected footpads. Mice were either non-immunized (•) or immunized with BCG (○) two months before challenge. All mice were infected in the footpad with 4 log10 CFU of M. ulcerans 98–912. At different times post-infection, total RNA from the footpad was extracted and the presence of mRNA for IL-4 (A), IL-10 (B), and IL-17 (C) was assessed by real-time PCR. Data points represent the mean ± SEM (n = 5–8) for each time point. Statistical significance was calculated with Student's t test (*p<0.05; **p<0.01; ***p<0.001). (TIF) [file pone.0033406.s001.tif]

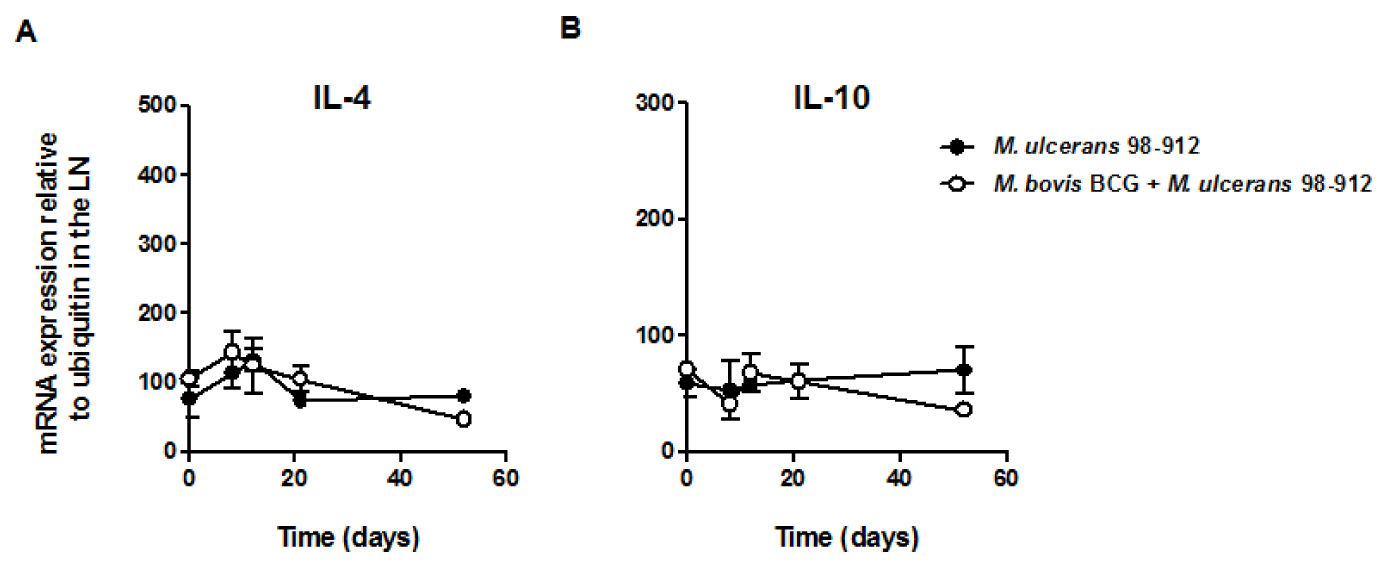

Supplement: Figure S2 — Vaccination with BCG induces an early Th1 cytokine profile in the DLN of M. ulcerans -infected mice. Mice were either non-immunized (•) or immunized with BCG (○) two months before challenge. All mice were infected in the footpad with 4 log10 CFU of M. ulcerans 98–912. At different times post-infection, total RNA from the popliteal lymph node was extracted and the presence of mRNA for IL-4 (A) and IL-10 (B) was assessed by real-time PCR. Data points represent the mean ± SEM (n = 5–8) for each time point. Statistical significance was calculated with Student's t test (*p<0.05). (TIF) [file pone.0033406.s002.tif]
